# Update on Myopia Risk Factors and Microenvironmental Changes

**DOI:** 10.1155/2019/4960852

**Published:** 2019-10-31

**Authors:** Valeria Coviltir, Miruna Burcel, Alina Popa Cherecheanu, Catalina Ionescu, Dana Dascalescu, Vasile Potop, Marian Burcea

**Affiliations:** ^1^Faculty of Medicine, “Carol Davila” University of Medicine and Pharmacy, Bd. Eroii Sanitari 8, Bucharest, Romania; ^2^Clinical Hospital of Ophthalmologic Emergencies (S.C.U.O), Alexandru Lahovari Square 1, Bucharest, Romania; ^3^Oftaclinic Clinic, Bd. Marasesti 2B, Bucharest, Romania; ^4^Department of Ophthalmology, University Emergency Hospital, Splaiul Independenței 169, Bucharest, Romania; ^5^CMDTAMP Clinic, Bucharest, Romania

## Abstract

The focus of this update is to emphasize the recent advances in the pathogenesis and various molecular key approaches associated with myopia in order to reveal new potential therapeutic targets. We review the current evidence for its complex genetics and evaluate the known or candidate genes and loci. In addition, we discuss recent investigations regarding the role of environmental factors. This paper also covers current research aimed at elucidating the signaling pathways involved in the pathogenesis of myopia.

## 1. Introduction

Myopia, also known as nearsightedness, is a common ocular disorder, which is considered a global problem because of the economic and social costs [1]. It affects typically school-age children and seems to progress the most between ages 8 and 15 due to the continuous growth of the eye during childhood [2–4].

The pathophysiology of myopia is multifactorial and is not yet completely understood. There are proofs that multiple genetic variations and environmental and lifestyle factors play an important role in the etiology of this disease [5]. Family linkage analysis, genome-wide association studies, and next-generation sequencing studies as well as a high correlation among monozygotic twins compared to dizygotic twins show that myopia has a genetic component [6–9].

On the contrary, studies have already shown the relationship between myopia and environmental factors such as near work, light exposure, lack of physical activity, and higher level of education revealing their major involvement in myopia development [10–12]. Although the genetic component has been widely studied, human population studies have revealed widely divergent prevalences of myopia among genetically similar populations in different environments, suggesting that development of myopia is controlled by both environmental and genetic factors [13–15].

New hypotheses suggest that the ethiopathogeny of myopia might also have an inflammatory component. Researchers revealed an increased prevalence of this refraction error in children with inflammatory diseases such as diabetes mellitus, juvenile chronic arthritis, uveitis, and systemic lupus erythematosus [16–19].

However, this is not without some controversy because many physiological and biochemical processes, not merely inflammation, are disturbed in these diseases; thus, the relationship between myopia and ocular and systemic inflammatory diseases is still debated in the recent literature. It is hypothesized that chronic hyperglycaemia and hyperinsulinaemia in a carbohydrate-rich diet could lead to overexpression of free insulin-like growth factor (IGF) level on one hand and underexpression of IGF-binding protein 3 level on the other hand that may result in scleral growth and implicit to juvenile-onset myopia [20].

Concerning the connection between diabetes mellitus and refractive error, there are variable results among studies that provided evidence of a myopic shift among young patients under 10 years with poor glycaemic control. However, in the older patients group there was no statistically significant difference in refraction [16, 21].

As for another autoimmune systemic disease, the association between myopia and juvenile chronic arthritis (JCA) has its limitations due to other biomechanical and biochemical factors that coexist with the inflammatory pathway. Thus, there is a higher incidence of myopic patients with JCA compared with a control group. These data could be explained by the effect of chronic inflammation on the sclera resulting in poor biomechanical properties of the connective tissue that could lead to myopization [22].

Lens-related myopization was found in inflammatory ocular conditions such as uveitis and Vogt–Koyanagi–Harada disease following corticosteroid therapy, respectively, through relaxation of zonular fibers and an increase of the lens' convexity caused by supraciliary exudation [17].

## 2. Inflammatory Profile in Myopia

It is postulated in the literature that myopia is usually a consequence of abnormal eye elongation, which is associated with scleral remodelation [23, 24]. It also has been shown that ocular size and refraction were regulated by extracellular matrix composition and its biomechanical properties [25].

Sclera is a fibrous connective tissue that consists of fibroblasts which play a key role in maintaining the extracellular matrix [25, 26]. In addition to fibroblasts, sclera comprises an extracellular matrix which consists of collagen fibrils (mainly type 1 collagen) and small amounts of fibril-associated collagens [27]. In myopic eyes, the scleral tissue undergoes constant thinning due to the reduced connective tissue synthesis and increased collagen 1 (COL1) degradation [28, 29].

Various morphological changes in the scleral extracellular matrix have been involved in myopia progression, besides the scleral thinning. All these changes are the result of biochemical and biomechanical signaling pathways showing a decreased amount of biomarkers for collagen and glycosaminoglycans [30].

Scleral fibroblasts are responsible for the expression of some proteins such as matrix metaloproteinase (MMP) and tissue inhibitor of matrix metalloproteinase (TIMP).

Taking into account that an animal model suggested an important role for MMPs in the development of experimental myopia, Hall et al. investigated the relation between myopia and variations in three genes coding for metalloproteinases. Their results suggested an overexpression of MMP 1, MMP 3, and MMP 9 that may contribute to the development of simple myopia [31]. MMPs are a type of enzymes that are responsible for the degradation of extracellular matrix proteins [32], tissue reconstruction [33, 34], and tissue vascularization during the inflammatory response [35] as well as for modulating scleral extensibility. More recent studies have provided evidence that MMPs are regulated by many cytokines and growth factors, including hs-CRP, tumor necrosis factor, and complement components [36–38]. In addition, MMPs are inhibited by tissue inhibitor of metalloproteinases (TIMPs) [32]. This complex (MMP-TIMP) is responsible for the integrity of the connective tissue and a normal wound healing after injuries [39].

Lin et al. demonstrated the presence of CC genotype in (TGF)-*β* codon 10 in patients with high myopia [40]. Other studies stated the involvement of TGF-*β* in scleral remodeling [28, 41]. It regulates the production of extracellular matrix, its turnover being the basic mechanism involved in axial length changes [42]. Researchers have reported that TGF-*β* modulates the level of MMP 2 throughout the activation of nuclear factor (NF)-*κ*B, which determines the production of inflammatory cytokines in fibroblasts such as TNF-*α* and IL-6 [43]. More than that, overexpression of TGF-*β* continues to activate expression of MMP2, which cleaves COL1 and becomes downregulated in a myopic eye [44, 45]. Li and colleagues revealed that a reduced expression of TGF-beta isoforms in the sclera is associated with a decreased synthesis of collagen and could be associated to an increased predisposition to pathological axial elongation [46].

TNF-*α* (tumor necrosis factor-alpha) is a transmembrane protein involved in systemic and local inflammation. It is produced by macrophages, lymphoid cells, and fibroblasts in response to bacterial products, IL-1, or IL-6. Recent evidence suggests that the inflammatory activity of the tumor necrosis factor family is more important than their role in apoptosis [47].

Such interactions between cells within the scleral extracellular matrix demonstrate changes in scleral biomechanical properties and scleral biochemistry, which subsequently lead to ocular elongation and thus a possible development of myopia [30].

In order to study the role of inflammation in myopia progression, Lin et al. investigated the expression of some proteins involved in inflammatory responses such as c-Fos, NF*κ*B, IL-6 (interleukin 6), and tumor necrosis factor-*α* (TNF-*α*). The study showed increased levels of these proteins in hamsters with myopia. They also found an increased expression of these proteins in eyes treated with lipopolysaccharide and peptidoglycan and a corresponding increase in myopia progression in hamsters. On the other side, there was a decrease in inflammatory protein expression and a corresponding decrease in myopia progression in hamsters treated with cyclosporine, an anti-inflammatory medication [48].

Wei et al. reported the theory that allergic inflammation of the eye would mediate the development of myopia. The study revealed that children with allergic conjunctivitis have a higher incidence and subsequent risk of myopia (2.35 times higher) compared to those without allergic conjunctivitis.

Moreover, they established an allergic conjunctivitis animal model to demonstrate the possible mechanisms underlying allergic inflammation as a risk factor of myopia. They found that the rats with allergic conjunctivitis have developed myopia (change in refractive error (RE) = −1.68 ± 2.52 D), whereas the rats in the control group did not (change in refractive error 1.07 ± 1.56 D).

In addition, the axial lengths of allergic conjunctivitis eyes were significantly longer (change in axial length = 0.27 ± 0.12 mm) than those of the control eyes (0.14 ± 0.09 mm).

In normal subjects, activation of the complement system is well regulated in the human body in order to avoid overstimulation and damage resulting from inflammation [45].

Long et al. discovered in 2013 in patients with pathologic myopia the overexpression of C3 and CH50 levels that suggest complement activation-induced inflammation may play an important role in the pathogenesis of myopia [49].

To confirm the relationship between inflammation and myopia, an animal model was established. Gao et al. published statistically significant increased levels of C1q, C3, and C5b-9 in the sclera of guinea pigs with myopia showing that activation of the complement system may induce extracellular matrix remodeling and development of myopia subsequently [50].

Recent studies evidence the correlation between the development and progression of myopia and activation of the complement system. In a meta-analysis of eight transcriptome databases for lens-induced or form-deprivation myopia, Riddell and Crewther found that the complement system is strongly activated in chick models of myopia [51].

## 3. Contribution of Oxidative Stress to the Development of Myopia

Oxidative stress begins to gain importance in the pathogenesis of glaucoma, age-related macular degeneration, dry eye syndrome, keratoconus, and myopia [52–56]. Oxidative stress results from the imbalance between free radical production on one hand and antioxidant defense mechanisms on the other [57]. It determines oxidative damage by altering cellular functions in addition to causing inflammation and cell death [58, 59].

Numerous studies have shown that elements such as zinc (Zn), copper (Cu), selenium (Se), manganese (Mn), *α*-tocopherol (vitamin E), ascorbic acid (vitamin C), glutathione (GSH), and *β*-carotene play an important role in the antioxidative processes [60–62] and in biochemical rebuilding of the sclera [63, 64].

A key role of the retina is to maintain an adequate oxygen supply. Under normal physiological conditions, metabolism of oxygen produces reactive oxygen species, one of the major contributors of oxidative stress [65].

Retinal tissue has the highest oxygen consumption in the body, thus determining the overexpression of ROS [57]. As ROS elevates, it may impair blood flow to the retina, which in consequence could lead to an increased level of oxidative stress [66]. Also, the continuous light exposure of the retina generates high amounts of ROS. These facts, the massive oxygen consumption and the light exposure, could be important conditions to argument the correlation between oxidative stress and myopia [57].

In order to predict the oxidative stress status in myopic patients, Kim et al. measured aqueous humor levels of 8-OHdG in 15 highly myopic eyes and 23 control eyes, taking into consideration that 8-OHdG is one of the most widely analyzed biomarkers regarding cellular oxidative stress [67]. They reported that 8-OHdG level was lower in the highly myopic group compared to the control group, a result that could indicate a reduced metabolic activity in myopic eyes which might bring on a decrease in oxidative stress level [68].

Taking into consideration that Zn insufficiency leads to oxidative damage [69], Fedor and coworkers investigated serum zinc and copper concentration as well as Cu/Zn ratio in the serum of children and adolescents with moderate and high myopia in order to assess the relationship between myopia and oxidative stress. They observed significantly lower serum concentration of Zn as well as significantly higher Cu/Zn ratio in myopic patients in comparison to the control group. Hence, these results may imply an association between insufficiency of these antioxidant microelements and the development of the myopia. Also, the higher ratio Cu/Zn in the study group indicates the disturbances of antioxidative mechanisms in patients with myopia [70].

Genetic studies have demonstrated that myopia is related with various growth factors, such as HGF (hepatocyte growth factor), which is capable of protecting the antioxidant system [71] by activating antioxidant genes such as catalase [72]. Based on the recent literature, it plays a key role in preventing oxidative damage; hence, it could become an important concern in myopia treatment in the future [57].

## 4. Recent Advances in Genetics of Myopia

It is known that myopia is a complex disease resulting from the interplay between multiple environmental and genetic risk factors. The studies mentioned below will highlight the most relevant conclusions concerning the topic of genetics in myopia.

The wide variability of the prevalence of myopia in different ethnic groups is an important aspect that supports its genetic component [73]. The prevalence of myopia is higher in Asians −70–90% compared with 30–40% in Americans and Europeans [74, 75]. Even if ethnicity has a major contribution to the prevalence of myopia, the literature shows widely divergent prevalences of myopia among genetically similar populations in different environments. For example, Rose and colleagues compared the prevalence and risk factors for myopia in children of Chinese ethnicity in Sydney and Singapore. They found a lower prevalence of myopia in Sydney, 3.3% versus 29.1% in Singapore (*p* < 0.001) which was associated with increased hours of outdoor activities (13.75 versus 3.5 hours per week; *p* < 0.001) [76].

So, whether myopia is due to interethnic differences in the genetic predisposition or cultural influences is still questionable.

In order to better understand the genetic background of myopia, several studies comparing monozygotic and dizygotic twins have been conducted, taking into consideration that monozygotic twins are identical in genetic material, while dizygotic twins share 50% of their genetic material. In this regard, Karlsson et al. found that the heritability of myopia was greater in monozygotic twins (95%) compared with 29% in dizygotic twins [77]. This finding was confirmed by other studies, in which the heritability in monozygotic twins varied from 55% to 94% [6, 7, 78, 79].

Also, monozygotic twins, who are much similar, phenotypically, than dizygotic twins, have a higher chance to have the same activities and hobbies, so the environmental changes could also have a high impact in myopia development and progression.

Besides the twin studies which underline the important role of genetic factors in the development of myopia, familial aggregation has also provided strong evidence to support the involvement of genetic factors in the pathogenesis of myopia [80–83].

It is considered that while common myopia is generally transmitted as a complex trait, high myopia can be transmitted either as a complex trait or a Mendelian trait, including autosomal dominant (AD), autosomal recessive (AR), and X-linked recessive (XL) inheritance [84].

Mutti et al. evaluated the interaction between near work and parental myopia to test the hypothesis of inherited susceptibility. They reported that myopia appears to be more frequent in children whose both parents are myopic (32.9% versus 6.3% in children whose both parents are emmetropic), with no evidence being found to support the hypothesis that children with myopic parents can inherit a susceptibility to the environment [85].

In supporting this finding, the study conducted by Ip et al. in 2007 reported that the proportions of myopia were 7.6% in children with no myopic parents, 14.9% in children with one myopic parent, and 43.6% in children whose both parents are myopic [81].

Additional evidence supporting the role of genetics in the development of myopia includes the wide variability of the myopia-associated genes. Recent genome-wide association studies (GWAS) have identified more than 20 myopia-associated loci that involved in neurotransmission (e.g., GRIA4), ion transport (e.g., KCNQ5, CD55, and CHNRG), retinoic acid metabolism (e.g., RDH5, RORB, and CYP26A1), extracellular matrix remodeling (e.g., LAMA2 and BMP2), and eye development (e.g., SIX4, PRSS56, and CHD7) [86, 87].

On the other hand, family-based linkage studies have revealed at least 12 myopia-associated loci, with MYP loci numbered according to their time of discovery. These loci were mapped in fewer than 5% of persons with high myopia. Thus, taking into account the high prevalence of high myopia in the general population, it is supposed that more loci and genes will be discovered [46].

To date, candidate gene association studies identified high myopia-associated genes such as collagen, type I, alpha 1 (COL1A1), transforming growth factor beta 1 (TGFB1), transforming growth beta-induced factor (TGIF), lumican (LUM), hepatocyte growth factor (HGF), myocilin (MYOC), paired box 6 (PAX6), and uromodulin-like 1 (UMODL1). However, further studies need to establish the causative mutations [88–95].

Tang et al. focused on PAX6 gene, that is, a gene involved in oculogenesis and has a role in the change of refractive power as well as in the change of axial length, and thus in myopia development or progression [96, 97]. The researchers investigated the association of the paired box gene 6 (PAX6) with different stages of severity of myopia to confirm whether the PAX6 gene is a genetic determinant only for higher grade myopia, or it has an impact also on a low-grade stage of myopia. They found that PAX6 is a genetic determinant for extreme myopia rather than lower grade myopia, suggesting that PAX6 could be involved in the development or progression into severe myopia, but could not impact the myopia onset [98].

Interestingly, the fact that some potential myopia-associated genes may be limited only to certain subtypes of myopia has been of great concern and research interest.

Recent genetic studies suggested that IGF-1 should be evaluated with caution as a candidate gene for myopia. Even if IGF-1 is involved in cellular growth and differentiation as well as in the apoptosis [99, 100], IGF-1 gene may not determine the susceptibility to high or very high myopia in Caucasians and Chinese [101]. This fact suggests that different single-nucleotide polymorphisms (SNPs) of the same gene may have different results in terms of their associations with myopia [30]. For example, HGF gene polymorphisms investigations reported that rs3735520 is associated with mild and moderate myopia, but not with high myopia, while rs2286194 could be related to high myopia. Also, TGFB 1 gene which encodes TGF-*β* presents similar phenomenon [102].

Another approach for the candidate gene screening relies on the investigation of the genes associated with myopic syndromes [46]. Sun et al. analyzed data from 298 patients with early-onset high myopia and verified mutations in all the genes responsible for systemic diseases accompanied by high myopia, in order to identify another candidate gene associated with myopia. The authors evidence the idea that early-onset high myopia, occurring before school age, is an ideal model for monogenic studies of high myopia because of the minimum influence of environment. Besides the already known genes associated with high myopia (SCO2, ZNF644, LRPAP1, SLC39A5, LEPREL1, and CTSH), they identified another candidate gene. For example, mutations in genes COL2A1 and COL11A1 associated with Stickler syndrome, CACNA1F associated with congenital stable night blindness, and RPGR associated with retinitis pigmentosa were predominantly discovered [103, 104].

In addition, Flitcroft et al. investigated polymorphisms located in and around genes known to cause rare genetic syndromes featuring myopia and found them to be overrepresented in GWAS studies of refractive error and myopia. They identified 21 novel genes (ADAMTS18, ADAMTS2, ADAMTSL4, AGK, ALDH18A1, ASXL1, COL4A1, COL9A2, ERBB3, FBN1, GJA1, GNPTG, IFIH1, KIF11, LTBP2, OCA2, POLR3B, POMT1, PTPN11, TFAP2A, and ZNF469) and several novel pathways (mannosylation, glycosylation, lens development, gliogenesis, and Schwann cell differentiation) potentially involved in myopia [105].

## 5. Environmental Background

While genetic factors play important roles in ocular refraction, it has been convincingly established that environmental factors have an essential impact on myopia development.

Up to now, lifestyle factors such as near work, light exposure, lack of physical activity, and higher level of education and urbanization have been shown to be involved in the etiopathogenesis of myopia [81, 85,106].

Near-work activities, such as reading, writing, computer use, and playing video games, are supposedly responsible for the high prevalences and progression rates of myopia [81, 107].

The Sydney Myopia Study reported that near work such as close reading distance (<30 cm) and continuous reading (>30 minutes) independently increased the odds of having myopia (odds ratio 2.5; 95% CI 1.7–4; *p* < 0.0001, respectively; odds ratio 1.5; 95% CI 1.05–2.1; *p*=0.02) [108].

In 2013, French et al. reported on children in the Sydney Adolescent Vascular and Eye Study and noted that children who became myopic performed significantly more near work (19.4 vs. 17.6 hours; *p*=0.02) compared with children who remained nonmyopic [109].

Huang et al. highlighted, in a recent systematic review and meta-analysis, that near-work activities were related with higher odds of myopia (odds ratio 1.14; 95% CI 1.08–1.20) and that the odds of myopia increased by 2% (OR: 1.02; 95% CI 1.01–1.03) for every one diopter-hour more of weekly near work [110].

In contrast, there are studies reporting that near work is not associated with faster rates of myopia progression [85, 111–113].

Therefore the relationship between near work and myopia is complex and needs to be investigated.

On the other hand, several recent epidemiological studies suggest that greater time spent outdoors might have a protective effect against myopia development and progression [114–116].

The mechanism of this association is still poorly understood, but in the literature there are two theories proposed: One of them is the “light-dopamine theory” which highlights that increased light intensity during time spent outdoor protects against myopia by the increased release of dopamine [114, 117–119].

As for the second one, “vitamin D theory” hypotheses that the increased ultraviolet light triggers the stimulation of vitamin D production, with a direct protection against myopia development [120–123].

The recently published meta-analysis by Tang et al. reported that lower 25-hydroxyvitamin D (25(OH)D) concentration is associated with increased risk of myopia (AOR: 0.92; 95% CI 0.88–0.96; *p* < 0.0001) [124].

Also, the recent Guangzhou randomized trial reported a significant opposed relationship between outdoor activities and incidence of myopia showing that the increase of time spent outdoor determines a relative reduction of 23% of the incidence of myopia [115].

## 6. Conclusions

Nowadays, myopia is considered a major public health concern. The pathogenesis of myopia is not yet completely understood. We can state that myopia is a complex disease with a multitude of factors including genetic, environmental (external), and microenvironmental components.

We now know that myopia has a genetic component and a number of genes and candidate loci being identified as related to the disease, but environmental factors such as high level of education, prolonged near work, light exposure, and lack of outdoor activities seem to have a very important role. Many studies have shown the role of the inflammatory process in myopia and the expression of some proteins related to changes in collagen fibers, scleral thinning, and axial length elongation.

After reviewing the most relevant and recently published results, we emphasize that the complete mechanism underlying the abnormal physiological changes in the development and progression of myopia would be better understood if the investigation is conducted at the cellular and molecular level. Thus, further studies are required.

A number of genes and candidate loci have been revealed, and as we elucidate, understanding the underlying cause of myopia could help identify potential targets for therapeutic intervention and slow or prevent progression and myopic complications.
